# Intra-operative hydroxyethyl starch is not associated with post-craniotomy hemorrhage

**DOI:** 10.1186/s40064-015-1126-0

**Published:** 2015-07-16

**Authors:** James A Feix, C Andrew Peery, Tong J Gan, David S Warner, Michael L James, Ali Zomorodi, David L McDonagh

**Affiliations:** Department of Anesthesiology, Duke University Medical Center, Durham, NC USA; Department of Neurosurgery, Duke University Medical Center, Durham, NC USA; Department of Neurology, Duke University Medical Center, Durham, NC USA; Department of Anesthesiology, Stony Brook University, Stony Brook, NY USA; Departments of Anesthesiology, Neurology, & Neurosurgery, University of Texas Southwestern, 5323 Harry Hines Blvd., Dallas, TX 75390-9068 USA

**Keywords:** Colloid, Hydroxyethyl starch, Hemorrhage, Neuroanesthesia, Craniotomy, Neurosurgery

## Abstract

**Background:**

Intraoperative intravascular volume expansion with hydroxyethyl starch-based colloids is thought to be associated with an increased risk of post-craniotomy hemorrhage. Evidence for this association is limited. Associations between resuscitation with hydroxyethyl starch and risk of repeat craniotomy for hematoma evacuation were examined.

**Methods:**

Using a retrospective cohort of neurosurgical patients at Duke University Medical Center between March 2005 and March 2012, patient characteristics were compared between those who developed post-craniotomy hemorrhage and those who did not.

**Results:**

A total of 4,109 craniotomy procedures were analyzed with 61 patients having repeat craniotomy for post-operative hemorrhage (1.5%). The rate of reoperation in the group receiving 6% High Molecular Weight Hydroxyethyl Starch (Hextend^®^) was 2.6 vs. 1.3% for patients that did not receive hetastarch (P = 0.13). The reoperation rate for those receiving 6% hydroxyethyl Starch 130/0.4 (Voluven^®^) was 1.4 vs. 1.6% in patients not receiving Voluven (P = 0.85).

**Conclusions:**

In this retrospective cohort, intra-operative hydroxyethyl starch was not associated with an increased risk of post-craniotomy hemorrhage.

## Background

Hydroxyethyl starch (HES) based colloids have gained popularity in clinical anesthesia practice due to their effective intravascular volume expansion (Kozek-Langenecker 2005). Recently, there have been studies warning providers to limit the volume infused because of concerns for platelet dysfunction and increased bleeding (Kozek-Langenecker 2005; Westphal et al. 2009; Myburgh and Mythen 2013; Hartog et al. 2011), based on in vitro coagulation abnormalities (Avorn et al. 2003; Wilkes et al. 2001; Strauss 1981). For example, in a porcine model of liver trauma HES has been shown to provoke uncontrolled hemorrhage (Zaar et al. 2009). Similarly, in a small prospective randomized trial of 40 patients undergoing major surgery the impact of HES on coagulation was apparent, as HES reduced clot strength and increased perioperative hemorrhage by more than 50% (Rasmussen et al. 2014). Even when used at less than the recommended maximum dose, HES use has been associated with increased postoperative bleeding and transfusion in patients undergoing heart surgery requiring cardiac bypass (Avorn et al. 2003; Wilkes et al. 2001). There have also have been small studies associating the use of HES with intracranial bleeding and coagulopathy in the setting of subarachnoid hemorrhage (Damon et al. 1987; Knutson et al. 2000). The mechanisms causing this acquired coagulopathy include qualitative platelet dysfunction, decreases in Factor VIII/von Willebrand’s factor complexes (sometimes referred to as “Acquired von Willebrand’s disease-Type 1”), and qualitative disruption of fibrin clots (Trumble et al. 1995; Jonville-Bera et al. 2001).

The cranium is a closed compartment where small volumes of blood can cause clinical, even life-threatening, symptoms. Previous studies show that 1–2% of post-craniotomy patients have symptomatic hemorrhage requiring intervention (Treib et al. 1999; Field et al. 2001). We aimed to determine whether intraoperative HES administration, compared with no HES, was associated with post-craniotomy hemorrhage requiring reoperation in a large retrospective cohort.

## Results

**Figure 1:**
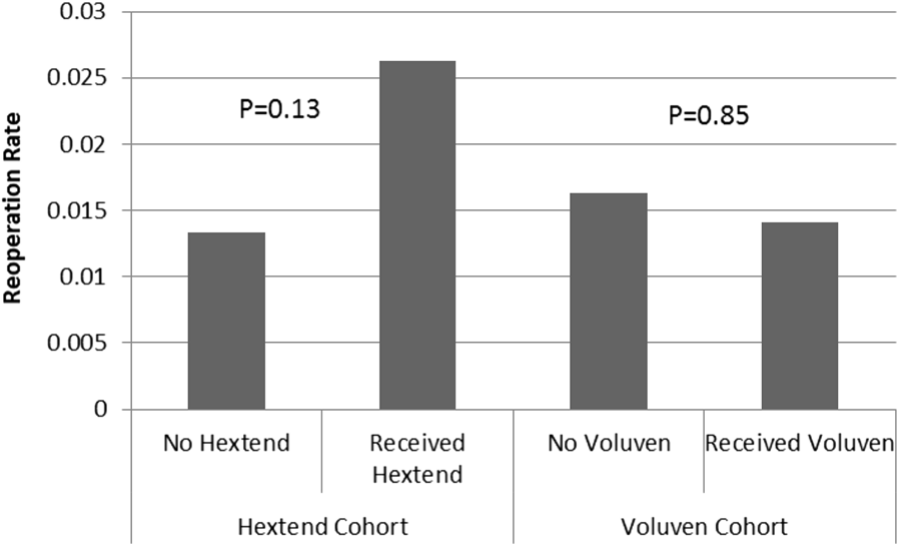
Comparison of reoperation rates (for postcraniotomy hemorrhage) for patients who did or did not receive HES products.

Out of a total of 4,109 craniotomy procedures, 61 patients underwent repeat craniotomy for post-operative hemorrhage. The overall reoperation rate for postoperative intracranial hemorrhage was 1.5%. The mean ± SD estimated blood loss was 331 ± 415 mL (Range 5–7,200 mL) and the average volume of crystalloid administered was 2,704 ± 1,863 mL (Range 75–20,200 mL). HES was used in 334 operations (8.1% of cases), including Hextend^®^ use in 190 cases (5.0%) and Voluven^®^ in 144 cases (2.8%) (Figure 1). The average volume of Hextend^®^ administered was 635 ± 286 mL (Range 100–1,600 mL) and the average volume of Voluven^®^ was 725 ± 350 mL (Range 250–2,000 mL).

Between March 2005 and March 2010, when Hextend^®^ was in use, a total of 2,883 craniotomies were performed. Of these procedures, 41 (1.4%) patients had a repeat craniotomy for post-operative hemorrhage. The post-operative hemorrhage group was more likely to be male, to have a tumor or trauma as the reason for craniotomy, and to have received a greater volume of crystalloid and packed red blood cells compared with those who did not have a post-operative hemorrhage (Table 1). During this time, there was no significant difference in the proportion of patients who received Hextend^®^ among those who had a post-operative hemorrhage and those who did not (Table 1).

**Table 1:** Demographic and clinical characteristics of the March 2005–March 2010 Cohort

	N = 2,801	N = 41	p value
	No repeat craniotomy for post-operative hemorrhage	Repeat craniotomy for post-operative hemorrhage	
Mean age, years (SD)	51.0 (16.0)	54.6 (15.5)	0.7
Male, n (%)	1,322 (47.4)	25 (62.5)	0.06
ASA grades 1–2, n (%)	1,236 (46.9)	20 (50.0)	0.82
ASA grades 3–5, n (%)	1,152 (53.1)	20 (50.0)	0.82
E Cases, n (%)	328 (12.1)	2 (5.0)	0.18
Tumor, n (%)	1,393 (49.7)	32 (80.0)	<0.0001
Vascular, n (%)	305 (10.9)	6 (15.0)	0.23
Trauma/other, n (%)	1,104 (39.5)	2 (5.0)	<0.0001
Baseline coagulopathy, n (%)	58 (2.1)	1 (2.5)	0.85
Mean EBL, mL (SD)	279.1 (391.9)	540.2 (1,176.2)	0.16
Mean operation length, min (SD)	229.2 (120.6)	226.3 (159.5)	0.15
Mean crystalloid volume, mL (SD)	2,694.9 (1909.5)	3,620.7 (2,351.1)	<0.0001
Received plasmanate*, n (%)	420 (14.7)	8 (19.5)	0.38
Mean plasmanate* volume, mL (SD)	529.7 (352.9)	750 (353.6)	0.21
Received hextend, n (%)	185 (6.6)	5 (12.5)	0.15
Mean hextend volume, mL (SD)	635.4 (286.9)	650 (335.4)	0.89
Received PRBCs, n (%)	194 (6.9)	3 (7.5)	0.89
Mean PRBCs volume, mL (SD)	877.5 (901.5)	2,450 (1,603.9)	0.003
Received FFP, n (%)	82 (2.9)	3 (7.5)	0.09
Mean FFP volume, mL (SD)	660.2 (603.0)	1,227 (1,050.7)	0.12
Received platelets, n (%)	91 (3.2)	1 (2.5)	0.77
Mean platelets volume, mL (SD)	353.0 (208.6)	563 (–)	–

* Plasmanate is a purified plasma derivative consisting predominantly of human albumin.

**Figure 2:**
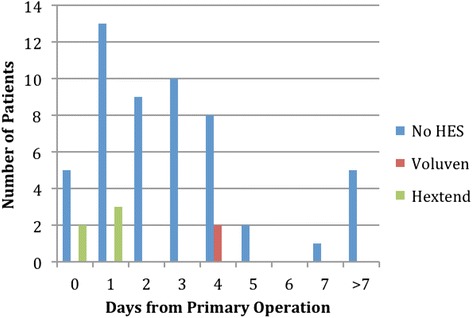
Timeline indicating when (post-operative day) patients returned for repeat craniotomy for hemorrhage.

Between March 2010 and March 2012, when Voluven^®^ was in use, a total of 1,287 craniotomies were performed. Of these procedures 20 (1.6%) patients had a repeat craniotomy for post-operative hemorrhage. The post-operative hemorrhage group was more likely to be older, to have a greater ASA Grade, and to have received packed red blood cells, fresh frozen plasma, and platelets compared to those who did not have a post-operative hemorrhage. During this time, there was no difference in the proportion of patients who received Voluven^®^ among those who had a post-operative hemorrhage requiring repeat craniotomy and those who did not (Table 2). Reoperation occurred most commonly on post-operative day 1 (Figure 2).

**Table 2:** Demographics and clinical characteristics of the March 2010 and March 2012 cohort

	N = 1,247	N = 20	p value
	No repeat craniotomy for post-operative hemorrhage	Repeat craniotomy for post-operative hemorrhage	
Mean age, years (SD)	51.6 (16.3)	61.8 (14.8)	0.007
Male, n (%)	632 (50.7)	9 (45.0)	0.61
ASA grades 1–2, n (%)	488 (39.1)	2 (10.0)	0.008
ASA grades 3–5, n (%)	734 (58.9)	18 (90.0)	0.008
E Cases, n (%)	112 (9.0)	1 (5.0)	0.53
Tumor, n (%)	784 (62.9)	13 (65.0)	0.72
Vascular, n (%)	288 (23.1)	3 (15.0)	0.39
Trauma/other, n (%)	175 (14.0)	4 (20.0)	0.45
Baseline coagulopathy, n (%)	15 (1.2)	0 (0)	0.62
Mean EBL, mL (SD)	297.5 (345.2)	236.1 (157.7)	0.11
Mean operation length, min (SD)	212.1 (109.9)	197.7 (138.5)	0.65
Mean crystalloid volume, mL (SD)	2,390.5 (1,482.8)	2,683.8 (1,494.2)	0.39
Received plasmanate*, n (%)	202 (16.2)	2 (10.0)	0.45
Mean plasmanate* volume, mL (SD)	510.4 (288.8)	625 (176.8)	0.53
Received voluven, n (%)	142 (11.4)	2 (10.0)	0.85
Mean voluven volume, mL (SD)	664.4 (287.7)	750 (353.6)	0.67
Received PRBCs, n (%)	72 (5.8)	3 (15.0)	0.08
Mean PRBCs volume, mL (SD)	832.2 (893.4)	350 (0)	<0.001
Received FFP, n (%)	39 (3.1)	3 (15.0)	0.003
Mean FFP volume, mL (SD)	583.3 (605.1)	539.67 (68.7)	0.67
Received platelets, n (%)	38 (3.1)	2 (10.0)	0.08
Mean platelets volume, mL (SD)	360.1 (226.7)	249 (69.3)	0.5

* Plasmanate is a purified plasma derivative consisting predominantly of human albumin.

## Discussion

Intraoperative HES use was not associated with reoperation for intracranial bleeding after craniotomy in our cohort. Similarly, Jian and colleagues found that HES was not a risk factor for post-craniotomy intracranial hematoma requiring surgery (Jian et al. 2014). The rate of reoperation for post-craniotomy intracranial hemorrhage in our cohort is similar to previously published rates (Field et al. 2001). Reoperation occurred most commonly on post-operative day 1, consistent with previous studies of post-operative hematoma evacuations (Jian et al. 2014; Kelly et al. 2011).

Our study has limitations. This is a secondary analysis performed using observational data collected at a single institution. We did not perform multivariate analyses to adjust for confounding variables. We also did not quantitatively examine dose–response between HES and blood loss because of the limited accuracy of retrospective HES dose and estimated blood loss data. We focused on repeat craniotomy for hemorrhage because it was an unambiguous endpoint. The study is underpowered to detect an increase in repeat craniotomies for post-operative hematoma in those exposed to HES, although our data indicate that the effect size is quite small. Our data may be helpful for any investigators planning prospective studies of HES in craniotomy patients. Based on our data, a future prospective study investigating the use of HES in craniotomy would need a sample size of 5,790 patients to have 80% power to detect an increase in repeat craniotomies for post-operative hematoma from 1.4% (HES group) to 2.1% (No-HES group) using a two-group Chi square test with a 0.05 two-sided significance level. This effect size is an odds ratio of 1.511.

## Conclusions

In conclusion, these data did not demonstrate an association between HES use during craniotomy and post-craniotomy hemorrhage. Whether low-volume intraoperative HES bolus dosing has adequate safety requires further study.

## Methods

After IRB approval was obtained, all craniotomy procedures at Duke University Medical Center between March 2005 and March 2012 were retrospectively reviewed using Duke Enterprise Data Unified Content Explorer (DEDUCE) and the electronic anesthesia records (Innovian, Drager, Telford, PA, USA). Patients undergoing re-operation within 30 days for intracranial hemorrhage were identified. If the operative note from the second operation indicated that the repeat craniotomy was to treat intracranial hemorrhage, such patients were placed in “Repeat Craniotomy for Postoperative Hemorrhage” group. All other craniotomy patients were placed in the “No Repeat Craniotomy for Postoperative Hemorrhage” group. Demographic information was obtained as well as relevant risk factors for post-operative bleeding including preoperative coagulation parameters, estimated blood loss, and operative site. HES use and other intraoperative fluids were identified from electronic anesthesia records associated with these cases. Of note, the attending neurosurgeons performing the majority of craniotomies as well as the intravenous fluids (normal saline and Ringer’s lactate) administered were consistent over the period of the study.

In March 2010, there was an institutional switch from Hextend^®^ (HES 6%/200/0.5) to Voluven^®^ (HES 6%/130/0.4). Our data set includes both HES products and analyzes data for each product separately.

### Statistical analysis

Means and standard deviations were reported for continuous variables. Medians were reported for skewed distributions of continuous variables. Proportions were reported for categorical data. Characteristics were compared using a 2 sample *t* test or Pearson’s Chi square test. All tests of significance were two-tailed and p values <0.05 were considered significant. The analysis was performed using SAS 9.2 (SAS, Cary, North Carolina).
